# Evaluation of photogrammetry for medical application in cardiology

**DOI:** 10.3389/fbioe.2023.1044647

**Published:** 2023-01-13

**Authors:** Giacomo Talevi, Luigi Pannone, Cinzia Monaco, Edoardo Bori, Ida Anna Cappello, Mara Candelari, Manon Wyns, Robbert Ramak, Mark La Meir, Ali Gharaviri, Gian Battista Chierchia, Bernardo Innocenti, Carlo de Asmundis

**Affiliations:** ^1^ Heart Rhythm Management Centre, Postgraduate Program in Cardiac Electrophysiology and Pacing, Universitair Ziekenhuis Brussel-Vrije Universiteit Brussel, European Reference Networks Guard-Heart, Brussels, Belgium; ^2^ BEAMS Department (Bio Electro and Mechanical Systems), Université Libre de Bruxelles, Brussels, Belgium; ^3^ Cardiac Surgery Department, Universitair Ziekenhuis Brussel-Vrije Universiteit Brussel, Brussels, Belgium

**Keywords:** photogrammetry, ECG imaging, CT scan, ablation, Brugada syndrome

## Abstract

**Background:** In the field of medicine, photogrammetry has played for long time a marginal role due to the significant amount of work required that made it impractical for an extended medical use. Developments in digital photogrammetry occurred in the recent years, that have steadily increased the interest and application of this technique. The present study aims to compare photogrammetry reconstruction of heart with computed tomography (CT) as a reference.

**Methods:** The photogrammetric reconstructions of digital images from ECG imaging derived images were performed. In particular, the ventricles of 15 patients with Brugada syndrome were reconstructed by using the free Zephyr Lite software. In order to evaluate the accuracy of the technique, measurements on the reconstructions were compared to patient-specific CT scan imported in ECG imaging software UZBCIT.

**Result:** The results showed that digital photogrammetry in the context of ventricle reconstruction is feasible. The photogrammetric derived measurements of ventricles were not statistically different from CT scan measurements. Furthermore, the analysis showed high correlation of photogrammetry reconstructions with CT scan and a correlation coefficient close to 1.

**Conclusion:** It is possible to reproduce digital objects by photogrammetry if the process described in this study is performed. The reconstruction of the ventricles from CT scan was very close to the values of the respective photogrammetric reconstruction.

## Introduction

Automatic digital photogrammetry is a surveying methodology that allows to build a three-dimensional model from digital photographs or videos. It offers an attractive alternative since it enables a radiation-free reconstruction for the patient. In the medical field, photogrammetry has played for a long time a marginal role due to the significant amount of work required. The developments of digital photogrammetry in the recent years increased the interest and the possible applications of this technique (Mitchell, 1995). In particular, the use of a photogrammetric approach allows the extraction of information related to the anatomical structure and the cardiac mapping under evaluation.

This can be used in cardiology to reconstruct the heart anatomy with the aim to build patient-specific models to guide invasive procedures, such as catheter ablation. The latter is based on targeting and elimination of an arrhythmogenic area in the heart, while sparing healthy tissue. The culprit region can be outlined by mapping techniques that allow to visualize the abnormal electrical activity of the region of interest (Pannone et al., 2022a; Pannone et al., 2022b; Pannone et al., 2022c).

Photogrammetry combines 3D volume acquisition and mapping in a single step. Indeed, often the information elaborated for mapping cannot be extracted from the mapping software but data can be used only as a marker of the presence of an arrhythmogenic area, as in the case of Brugada syndrome (BrS) (Cheniti et al., 2019). In this case the pathological area is an area of conduction delay in the absence of structural abnormalities.

Therefore, the aim of this study is to describe a novel photogrammetry procedure for the creation of a 3D model to plan and target the pathological area during epicardial ablation of Brugada syndrome.

## Materials and methods

### Non-invasive ECG imaging mapping

Fifteen consecutive patients with BrS underwent CT scan and non-invasive ECG imaging (ECGi) with CardioInsight™ Non-invasive 3D Mapping System (Medtronic Inc, Minneapolis, MN) technology. Previous studies in Brugada syndrome, showed that the ECGi mapping can delineate the pathological area and this is consistent with invasive mapping (Rudy and Messinger-Rapport, 1988; Zhang et al., 2016; Pappone et al., 2017; Salghetti et al., 2019). CardioInsight™ Non-invasive 3D Mapping System (Medtronic Inc, Minneapolis, MN) technology uses a vest with 252 electrodes applied to the patient; the unipolar electrocardiograms obtained from electrodes are projected on the 3D geometry derived from patient-specific CT scan. Using CardioInsight technology, activation time (AT), repolarization time (RT), and activation-repolarization interval (ARI) maps are obtained automatically.

The activation time for each unipolar electrogram was defined by the elapsed time between the onset of depolarization (QRS) and the maximum negative slope (maximum negative dV/dt) of the unipolar EGM. The activation delay was assessed through a post-analysis process with next-generation software (UZBCIT) developed for our Center by Medtronic Inc. that can provide a quantitative output to determine the pathological area in the right ventricle outflow tract. In particular, the region of interest was identified by the ECGi mapping examination, together with an experienced cardiologist, based on the late activation time.

The mapping software offers the possibility to visualize the volumetric mesh of the patient ventricle and electrical conduction. However, the anatomy and mapping information cannot be directly extracted from the software. Photogrammetry was developed to extract not only the anatomical structure but also the cardiac mapping information in a single 3D object. The studies involving human participants were reviewed and approved by Ethical Committee of UZ Brussel, Brussels, Belgium.

### Photographic report

**FIGURE 1:**
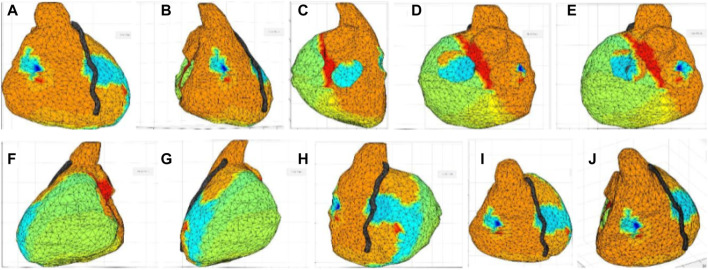
Image collection from the UZCBIT software. Starting images for photogrammetric reconstruction; **(A)** Image 30° angle with elevation angle on the x-axis of 0°, **(B)** Image 60° angle with elevation angle on the x-axis of 0°, **(C)** Image 120° angle with elevation angle on the x-axis of 0°, **(D)** Image 150° angle with elevation angle on the x-axis of 0°, **(E)** Image 210° angle with elevation angles on the x-axis of 0°, **(F)** Image 240° angle with elevation angle on the x-axis of 0°, **(G)** Image 300° angle with elevation angle on the x-axis of 0°, **(H)** Image 330° angle with elevation angle on the x-axis of 0°, **(I)** Image 30° angle with elevation angle on the x-axis of 30°, **(J)** Image 45° angle with elevation angle on the x-axis of 30°.

The ventricles reconstructed in the ECGi software (UZBCIT) were photographed from different angles, which corresponded to the frontal, right lateral, left lateral, posterior, and superior projections. The projections of the ventricles were virtually divided for the purpose of making the photographs. Photographs were made concentrically with respect to the z-axis at angles of 30°,45°, 60°, 90°, 120°, 135°,150°, 180°, 210°, 225°, 240°, 270°, 300°, and 330° with respect to three elevation angles on the x-axis of −30°, 0°, 30°. Thus, reproduction of each ventricle was performed with 45 photographs (Figure 1). Figure 1 shows how the photographs overlap. This is necessary so that the software, upon processing, easily recognizes similarities between photographs depicting adjacent areas (Figure 1).

In order to avoid errors and increase the precision of the results, the images were processed (pre-processed) using Masquerade software. The latter is a support application for the Zephir software, which allows to highlight the object to be reconstructed. In other words, Zephir will use for the reconstruction of the object, only the areas previously selected on Masquerade, ignoring the rest.

### Image processing by photogrammetry

**FIGURE 2:**
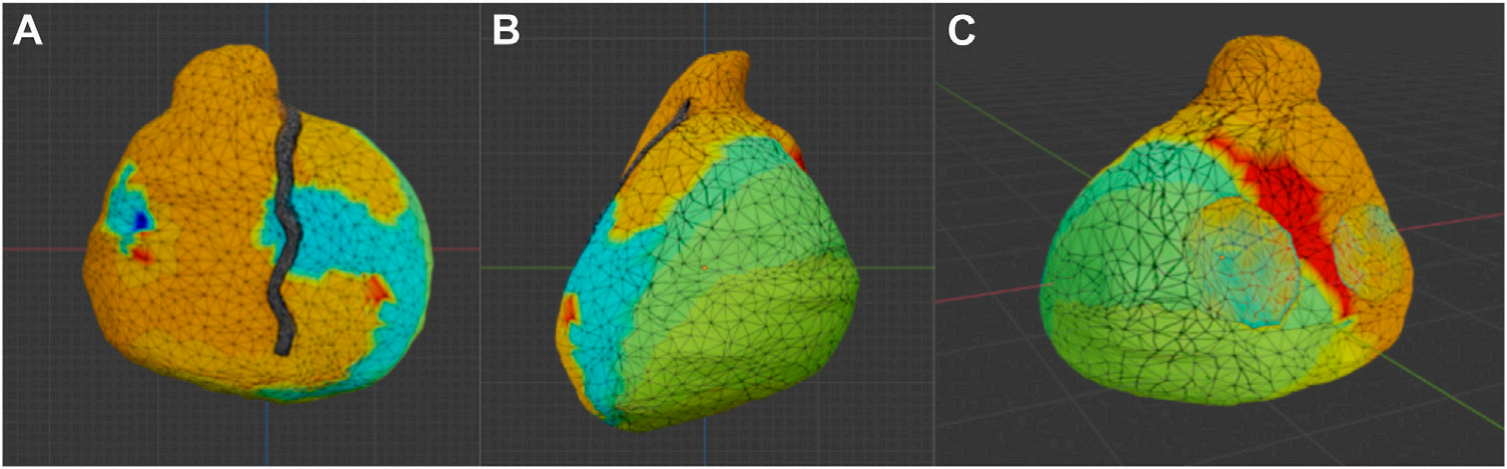
Model produced with photogrammetry. Photogrammetric reconstruction view in Blender. **(A)** Antero-posterior view; **(B)** Latero-lateral view, **(C)** Postero-anterior view.

The images were processed using a free version of the ZephirLite program (3DFlow^©^). The final 3D volume can be created by selecting a video or a series of images from which the software will extrapolate the dense point cloud and then form the 3D mesh (Figure 2).

Furthermore, the software enables the addition of a new calibration, and the modification of the automatic calibration performed previously. Afterwards, interactions are created with an interface that provides selection regarding camera orientation, category, and presets. The software works semi-automatically. Once the photos are loaded, the masks obtained with Masquerade are loaded and the parameters for reconstruction are set. The categories presets are divided into generic object, near objects, urban environment and human body; thus, based on the type of object to be processed, an optimal corresponding category can be chosen. Presets are divided into fast, default and deep analysis. They represent the search for a progressively larger number of common points between each image. For the purpose of our study, the reconstruction was obtained through the high-definition option for a generic object.

Once the scattered point cloud has been processed, if the results show that the object has been entirely reproduced as a point cloud, then the processing of the dense point cloud, the creation of the mesh, and ultimately the textured mesh can be performed. In the first place the software creates a cloud of scattered points, which are obtained automatically by locating the point in three or more images and subsequently a dense cloud is generated, to which a densification filter is applied to obtain a better visualization of the cloud itself. From this cloud, the software creates the mesh, and a filter is applied to the mesh for topology optimization. This processing can take about 2 h. At the end, the software allows the mesh to be exposed in the.obj format. This format is compatible with the 3D graphics software Blender.

### Image processing from CT scan and comparison with photogrammetry

In this study, a CT scan of the ventricles, employed for cardiac mapping visualization was used to obtain a comparison for accuracy of photogrammetric reconstruction. The CT scan was performed with 64 slices image acquisition and 360° rotations. After CT scan images were imported in the ECGi proprietary software and the selection of the region of interest for 3D reconstruction (ventricles), was performed manually, by proceeding to the segmentation of each component: ventricles, arteries and veins. The file processed was exported in .xml format and introduced into the UZBCIT software.

The UZBCIT software allows the simultaneous visualization of the ventricular volume and the mapping of the electrical activity of the heart (Figure 1). The software also enables the calculation of the area of specific sections in cm^2.

Once the photogrammetric model was created with Zephyr, it was imported into Blender 3.0 (Blender Foundation). In order to assess the level of accuracy of the Zephyr software’s photogrammetric reconstruction, the latter was compared to the 3D virtual model based on the CT scan of the UZBCIT software through the function Analyze > statistics > Area, present in Blender. Reference measurements are taken from the CT mesh, as it is considered the most accurate mesh (gold standard) (Covino et al., 1996). Comparisons were performed by calculating the area of the aorta and vena cava sections for both photogrammetric and CT-based models. The following measurements were defined: 1) U-aorta: area section of the aorta calculated on UZBCIT; 2) B-aorta: area section of the aorta calculated on Blender; 3) U-vena cava: area section of the vena cava calculated on UZBCIT; 4) B-vena cava: area section of the vena cava calculated on Blender.

These measurements have to be the same in the photogrammetry mesh. If the measurements of this mesh do not match those of the CT mesh, the mesh size can be increased or decreased through the “scale” tool by selecting the points of interest and increasing the size by dragging the mouse. Blender software can zoom in or out on the 3D mesh evenly throughout its volume. The 3D object is then enlarged according to each projection of its volume equally.

### Statistical analysis

Statistical analysis was performed using JASP software (GNU Affero GPL). Mean differences between meshes created by photogrammetric method and CT were used to assess either overestimation or underestimation of each method. If this is significantly different from zero, it is indicative of an overestimation or underestimation of one technique over the other. The mean difference was evaluated through the use of the paired *t*-test or Wilcoxon’s test on ranks, in the case of non-normal distributions. To evaluate the trend of the distributions, a descriptive analysis and a normality test with Shapiro Wilk’s test were performed; in addition, the Q-Q plot was performed as an auxiliary test of normality confirmation. Once the normality of the distributions was investigated, the Wilcoxon signed-rank test was performed, and to conclude, a correlation test base on a Bayesian information criterion was also performed.

## Results

Photogrammetric reconstruction of all 15 test subjects was successfully completed. The procedure took a total time of 2 h for each reconstruction. In detail, about 45 min were required for image acquisition, 50 min were required for preprocessing with Masquerade, and about 25 min were required for photogrammetric reconstruction from image loading to mesh export.

**FIGURE 3:**
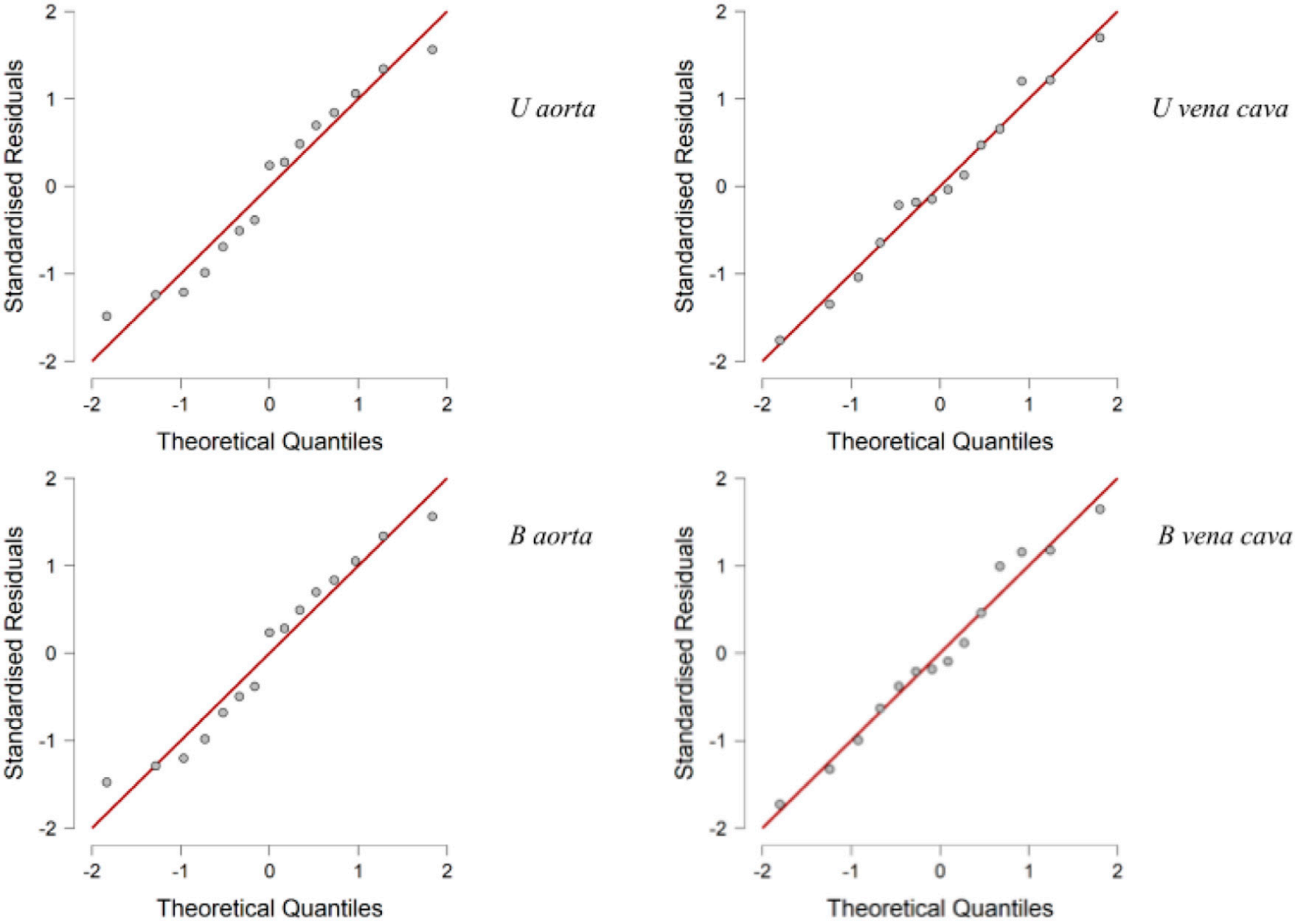
Q-Q Plots. Q-Q plot showing a distribution of data close to diagonal.

**FIGURE 4:**
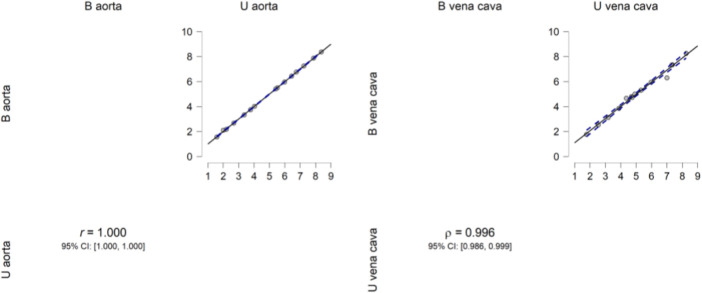
Pearsons’s correlation plot. Pearson’s correlation plot between the aorta and vena cava measurements from the two software programs. The data show high correlation.

**FIGURE 5:**
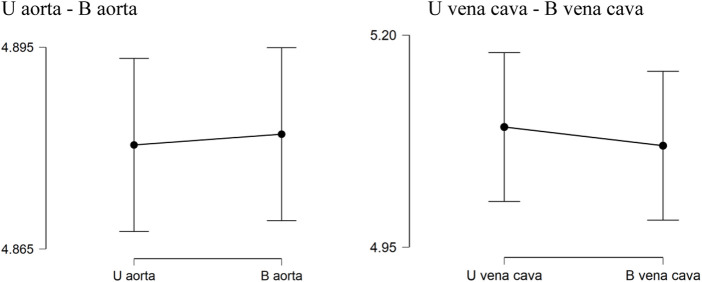
Descriptive Plots. U aorta-B aorta U vena cava-B vena cava. Graphical results obtained from descriptive analysis. The graphs show a not significant difference between the measurements obtained between CT and photogrammetry.

Descriptive analysis was performed. The values of the mean for the aorta data were 4.880 for U-aorta and 4.882 for B-aorta, while the mean values were 5.092 for U-vena cava and 5.070 for B-vena cava; with a Standard error of mean (SEM) of .557 for both aortas (U/B), .501 for U-vena cava and .514 for B-vena cava (Table 1). The analysis of the data showed a good correlation between the measurements acquired with photogrammetric reconstruction and CT images. Although the QQ plot (Figure 3) showed a distribution of data close to the diagonal, the test for normality showed a non-normal distribution (Table 2). Therefore, the Wilcoxon signed-rank test was performed, whose *p*-value was >.05 (Table 3), with a result of *p* = .348 for the aorta measurements and *p* = .638 for the vena cava measurements, respectively. This demonstrated a non-significant difference between photogrammetry and CT scan derived images. For further confirmation, a Pearson’s correlation test was also performed, with coefficient value proximal to one for both analysis; the graphs supported a strong correlation between the mesh reconstruction derived from CT and that derived from photogrammetry (Figure 4). The test showed for the section of the aorta a homogeneity of results. From the graph in Figure 5, it can be deduced that photogrammetry slightly overestimated the measurements compared with CT for the aorta; on the other hand, for the vena cava section, photogrammetry slightly underestimated the measurements compared with CT.

**TABLE 1:** Descriptive statistics.

	U aorta	B aorta	U vena cava	B vena cava
Valid	15	15	15	15
Missing	0	0	0	0
Mean	4.880	4.882	5.092	5.070
Std. Deviation	2.236	2.234	1.922	1.874
Std. Error of Mean	.577	.577	.501	.514

Table 1 shows the results of the descriptive analysis.

**TABLE 2:** Test of normality (Shapiro-Wilk).

			W	*p*
U aorta	-	B aorta	.670	<.001
U vena cava	-	B vena cava	.631	<.001

Table 2 shows the results of the normality test performed on the collected data. Significant results suggest a deviation from normality.

**TABLE 3:** Wilcoxon Signed-rank test.

Measure 1	Measure 2	W	Df	*p*
U aorta -	B aorta	77.000		.348
U vena cava	B vena cava	60.500		.638

Table 3 shows the results of the Wilcoxon rank-sum test performed on the collected data. The results show a *p*-value >.05.

## Discussion

Automatic photogrammetry allows the processing of 3D imaging. In the medical field, its application is still limited (Mitchell, 1995). This study performed virtual reproduction of cardiac mapping for digital dimensional assessment. Zephyr Lite (3DFlow^©^) software was used for 3D volume creation. Because of the use of this software, the realization of a three-dimensional mesh was possible, starting from jpg images. The accuracy of the reconstructions has been evaluated using 45 photographs collected concentrically with respect to the z-axis from different angles. The mesh generation process involves structure-from-motion (SfM) methods, which identifies common features of the images using the scale-invariant feature transform (SIFT) algorithm (Bruschweiler et al., 2003). This research compared the results of applying two three-dimensional acquisition techniques using patients with Brugada Syndrome who underwent to cardiac mapping with CardioInsight. (Cluitmans et al., 2018; Cheniti et al., 2019). The 15 reconstructions were feasible in a relatively short time. The purpose of this research was also to evaluate the potential of the photogrammetric technique to be employed in cardiology with several potential applications. In this paper CT scan was used as gold standard. Comparing CT with photogrammetry is important considering the limitations and advantages of both techniques (Markose et al., 2009; Donato et al., 2020). The accuracy of the photogrammetric technique was evaluated and the results obtained are very encouraging. Photogrammetric reconstruction measurements were very close to those obtained from measurements made on CT-based reconstructions. These results confirmed that the photogrammetric technique is useful for three-dimensional reconstruction of digital images.

Moreover, it is essential to consider the impact on results if the hereby presented procedure is not followed. If Masquerade is not used, since it is a reconstruction of a digital object, the Zephir software makes incomplete and erroneous reconstructions. On the other hand, the reconstruction of virtual objects has the advantage of not requiring any manipulation of the mesh once it has been created. The aforementioned manipulation, in classical cases, is due to the presence of the support in which the object is placed for image acquisition.

For current practical use it is important to select a myriad of polygons that compose the mesh, as it will be possible to manipulate the mesh with greater precision. In fact, the software develops a line between the mesh to be preserved and the area to be removed. This line will automatically follow the perimeter of the polygons that make up the mesh. The smaller the number of polygons, the more precise the line will be. By doing so, a more precise edge to the removed area can be defined. If the number of polygons is too low, the accuracy of the line will be reduced (Donato et al., 2020), thus causing possible errors. It is essential to control the number of polygons that make up the mesh also in order to obtain a more accurate result during the measurements. Therefore, the experience of the operator is of fundamental importance to obtain accurate and reliable results.

CT will always be irreplaceable for the visualization of the structures within the area of interest; however, photogrammetry represents an excellent tool for the study and analysis of the surface also in the case of reproduction of digital objects. BrS is a primary arrhythmia syndrome with no structural changes. However, electrical changes (conduction delay) occur and they can be investigated with ECGi (Pannone et al., 2022b). One disadvantage of standard imaging techniques as CT scan or photogrammetry is that neither CT scan alone, nor photogrammetry alone can evaluate the electrical abnormalities in BrS but ECGi can. Photogrammetry cannot image the electrical changes in BrS on its own but it has an important clinical role in extracting the relevant geometrical and electrical information from an ECGi map, making these data available for a 3D surgical guide building. Based on the feedback from an experienced cardiologist (C.d.A.) and an experienced cardiothoracic surgeon (M.L.M.), photogrammetry was considered feasible and of major clinical impact to extract relevant data from ECGi maps.

## Conclusion

It is possible to reproduce digital objects by photogrammetry if the process described in this study is performed. The reconstruction of the ventricles from CT scan was very close to the values of the respective photogrammetric reconstruction. Future studies should be performed, with a larger number of samples and on different pathologies. In particular, assessing the reproducibility of the described approach for a 3D model in a large cohort is eagerly awaited. Future research is needed to test mechanical properties and further assess biocompatibility of materials for a 3D surgical guide. The response of the material to ablation should be also evaluated.

## Data Availability

The original contributions presented in the study are included in the article/Supplementary Material, further inquiries can be directed to the corresponding author.
